# Hematinic deﬁciencies in patients with recurrent aphthous stomatitis: variations by gender and age 

**DOI:** 10.4317/medoral.21885

**Published:** 2018-02-25

**Authors:** Zhe-Xuan Bao, Jing Shi, Xiao-Wen Yang, Li-Xin Liu

**Affiliations:** 1 First clinical medical school of Shanxi Medical University, Taiyuan, Shanxi, China; 2 Department of Oral Medicine, Shanxi Provincial People’s Hospital, Taiyuan, Shanxi, China; 3 Department of Hospital Infection Control, Shanxi Provincial People’s Hospital; Taiyuan, Shanxi, China; 4 Department of Gastroenterology and Hepatology, The First Clinical Hospital of Shanxi Medical University, Taiyuan, Shanxi, China

## Abstract

**Background:**

The aim of this study was to evaluate the association between hematinic deficiencies and recurrent aphthous stomatitis (RAS).

**Material and Methods:**

517 RAS patients and 187 healthy controls were enrolled in the present study. Hematinic deficiencies, including serum ferritin, folic acid, and vitamin B12 deficiencies were assessed for each participant. Gender and age were taken into account and the collected data were statistically analysed.

**Results:**

Compared with the healthy controls, a significantly higher overall frequency of hematinic deficiencies was found in RAS patients (*p*<0.001). When gender and age were taken into account, significant differences in hematinic deficiencies were observed among RAS patients. Serum ferritin deficiency was much more common in young and middle-aged female RAS patients (age<60). Serum folate deficiency and serum vitamin B12 deficiency were both much more common in the young adult group of male RAS patients (21–40 years of age). Logistic regression analysis revealed that both gender and age have significant correlation with the presence of hematinic deficiencies in the RAS patients.

**Conclusions:**

Significant variations in hematinic deficiencies were demonstrated in RAS patients across different genders and age groups. We suggest that further studies on the hematinic deficiencies of RAS patients should take into account the gender and age of participants.

** Key words:**Recurrent aphthous stomatitis, hematinic deﬁciencies, gender, age.

## Introduction

Recurrent aphthous stomatitis (RAS), characterized by recurrent ulcerations limited to the oral mucosa, is one of the most common oral mucosal disorders, affecting approximately 20% of the general population and reaching as high as 60% in specific areas or populations (1-3). Conventionally, RAS is classified into three clinical forms: minor, major and herpetiform ulcers according to its clinical characteristics, while more than 80% of patients exhibiting the minor form (1,4). Although RAS has a good long-term prognosis, the lesions associated with the condition are excruciating and may interfere with chewing, speaking, and swallowing (1).

Despite extensive investigations, the definite etiology and pathogenesis of RAS have not been well recognized (1-6). Hematinic deficiencies, including a lack of ferritin, folate, or vitamin B12 have been proposed as possible etiologic factors. However, the conclusions of existing studies are still inconsistent, and many contradictory results have been reported (2,7-17). Possible reasons for this include differences in race/ethnicity, geographic, and dietary characteristics among RAS patients (1,2,6). Furthermore, despite a high incidence in the general population, RAS patients have usually been considered in most studies simply as a single group, and the number of patients included has been quite limited. Although the significant influences of gender and age on hematinic deficiencies have been well studied and validated in the hematological field (18,19), these views still have not been fully evaluated and applied in the association between RAS and hematinic deficiencies. Therefore, further studies are necessary to determine whether the hematinic deficiencies of RAS patients vary significantly in different gender and age groups.

The present study, in which gender and age were taken into account in a larger number of RAS patients, aimed to explore the hematinic deficiencies in RAS patients more accurately. Important implications for clinical practice are also discussed.

## Material and Methods

A total of 517 patients with diagnosis of minor RAS were evaluated and enrolled in this study at the Department of Oral Medicine, Shanxi Provincial People’s Hospital, China from June 2013 to August 2016. The diagnosis of RAS is typically established from the history and clinical presentation (4). Patients with Behcet’s syndrome, HIV, Oral cancer, gastrointestinal diseases and other relevant systemic diseases were excluded from this study (6,16,20). The RAS patients group consisted of 250 males and 267 females, ranging in age from 8 to 84 years. In addition, 187 gender- and age-matched healthy control subjects (89 males and 98 females) with no history of RAS were also examined. No subject in the control group exhibited any oral mucosal diseases or relevant systemic diseases. Participants were divided into four groups according to age, with cut-off points at 20, 40, and 60 years. Medical history and other related information of each participant were recorded in detail, and a careful clinical examination of oral mucosa was performed by the same dentist. The study was approved by the Shanxi Provincial People’s hospital ethics committee.

After obtaining the written informed consent, blood samples were collected from each participant after an overnight fast. The clinical laboratory responsible for the blood tests was also blinded to the group allocation. Serum vitamin B12 levels were measured using an Access Vitamin B12 Assay Kit (Beckman Coulter, Brea, CA). The accepted normal serum vitamin B12 level is 180–914 ng/L. Serum ferritin were measured using an Access Ferritin Assay Kit (Beckman Coulter, Brea, CA). The accepted normal serum ferritin level for females is 11.0–306.8 ng/ml, and for males is 15–336.2 ng/ml. Serum folate levels were measured using an Access Folate Assay Kit (Beckman Coulter, Brea, CA). The accepted normal serum folate level is 4.0–18.7 ng/ml. Serum vitamin B12, ferritin, and folate deficiencies were defined as serum levels below their cut-off values in all cases.

- Statistical analyses

Statistical analysis was evaluated using SPSS software, version 18.0 (Chicago, Illinois, USA). The frequencies of hematinic deficiencies between the groups were compared using chi-square test and fisher’s exact test was applied if the observed frequency was less than 5. Least significant difference (LSD) method for multiple comparisons was adopted for pairwise comparisons when necessary. Logistic regression analysis was conducted to assess statistical correlation between the two factors (age and gender) and the presence of hematinic deficiencies (ferritin, folate and vitamin B12 deficiency) in the RAS patients group and healthy control group, respectively. A *p*-value less than 0.05 was considered as statistically significant.

## Results

1. Hematinic deﬁciencies in RAS patients compared with healthy controls

Based on the normal laboratory values, the overall frequency of hematinic deficiencies was 45.6% (236/517) in all RAS patients versus 27.8% (52/187) in healthy controls, with highly significant difference (*p*<0.001). The rate of serum vitamin B12 deficiency in RAS patients was significantly higher than that in healthy controls (*p*<0.001). However, no statistically significant differences were found in the rate of serum ferritin deficiency or serum folate deficiency between RAS patients and healthy controls (*p*=0.845 and *p*=0.343, respectively). Furthermore, a combination of deficiencies (more than one deficiency) was also much more common in RAS patients compared with healthy controls (*p*=0.013).

2. Hematinic deficiencies in RAS patients according to gender and age

Statistically significant differences in hematinic deficiencies were found between male and female RAS patients. Serum ferritin deficiency was much more common among female patients (*p*<0.001,  Table 1). By contrast, serum folate deficiency and serum vitamin B12 deficiency were both much more common in male patients (*p*=0.001 and *p*=0.043, respectively). Considering age only (irrespective of gender), significant differences in hematinic deficiencies were also revealed among age subgroups of the RAS patients, except the serum vitamin B12 deficiency ( Table 1). For comparison, the same statistical evaluations were conducted in the healthy control group. No ferritin deficiency was found in healthy males and the rate of serum ferritin deficiency in healthy females was only 6.1% (6/98), the statistical difference was significant (*p*=0.018,  Table 1). However, neither serum folate deficiency nor serum vitamin B12 deficiency was detected significant differences between healthy males and healthy females and no significant difference of hematinic deficiencies was observed among the age subgroups in the healthy controls ( Table 1).

**Table 1:**
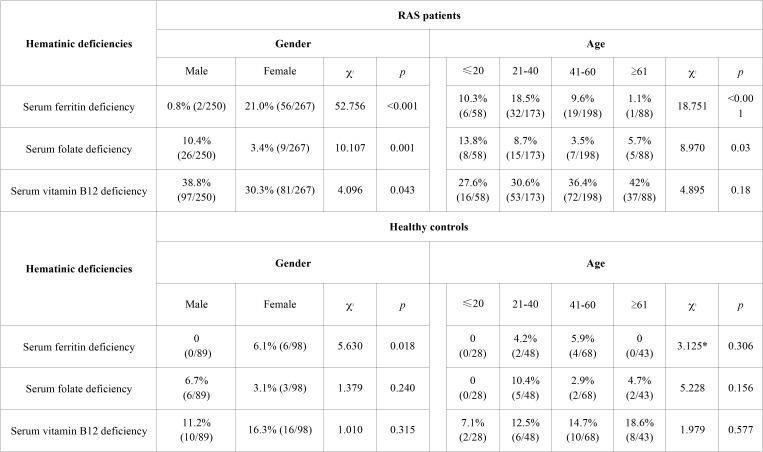
The respective statistical analysis of hematinic deficiencies in RAS patients and healthy controls according to gender and age.

3. Hematinic deﬁciencies in RAS patients compared with healthy controls with respect to gender and age

When gender and age were taken into account simultaneously, statistically significant changes of hematinic deficiencies were also observed in RAS patients ( Table 2). Serum ferritin deficiency was relatively uncommon in male and older female RAS patients (age ≥61), but much more common in younger females. Serum folate deficiency was more common in young and middle-aged male RAS patients. Meanwhile, serum vitamin B12 deficiency was also more common in the lower age groups of male RAS patients, especially in the young adult group (21–40 years of age) compared with female RAS patients.

**Table 2:**
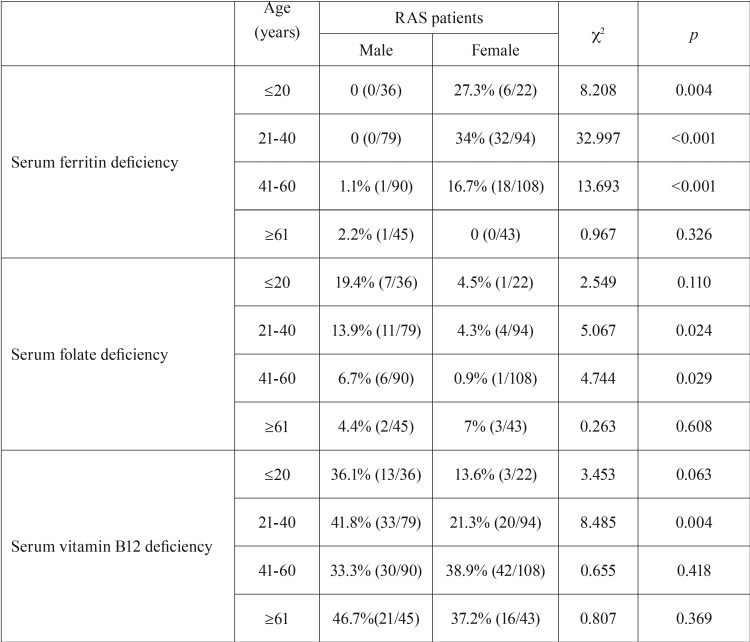
Significant variation in hematinic deficiencies among RAS patients according to gender and age.

Compared with healthy male controls, male RAS patients had a higher frequency of vitamin B12 deficiency in each age group, although the difference was statistically significant in the young adult and older group only (*p*=0.009 and *p*=0.001, respectively), with no statistical difference in the adolescent group and middle-aged group (*p*=0.131 and *p*=0.063, respectively). Regarding serum ferritin deficiency and serum folate deficiency, no significant differences were found between male RAS patients and healthy male controls. However, a borderline significant difference in rate of serum folate deficiency was found in the adolescent group (*p*=0.051). Detailed results are shown in  Table 3.

**Table 3:**
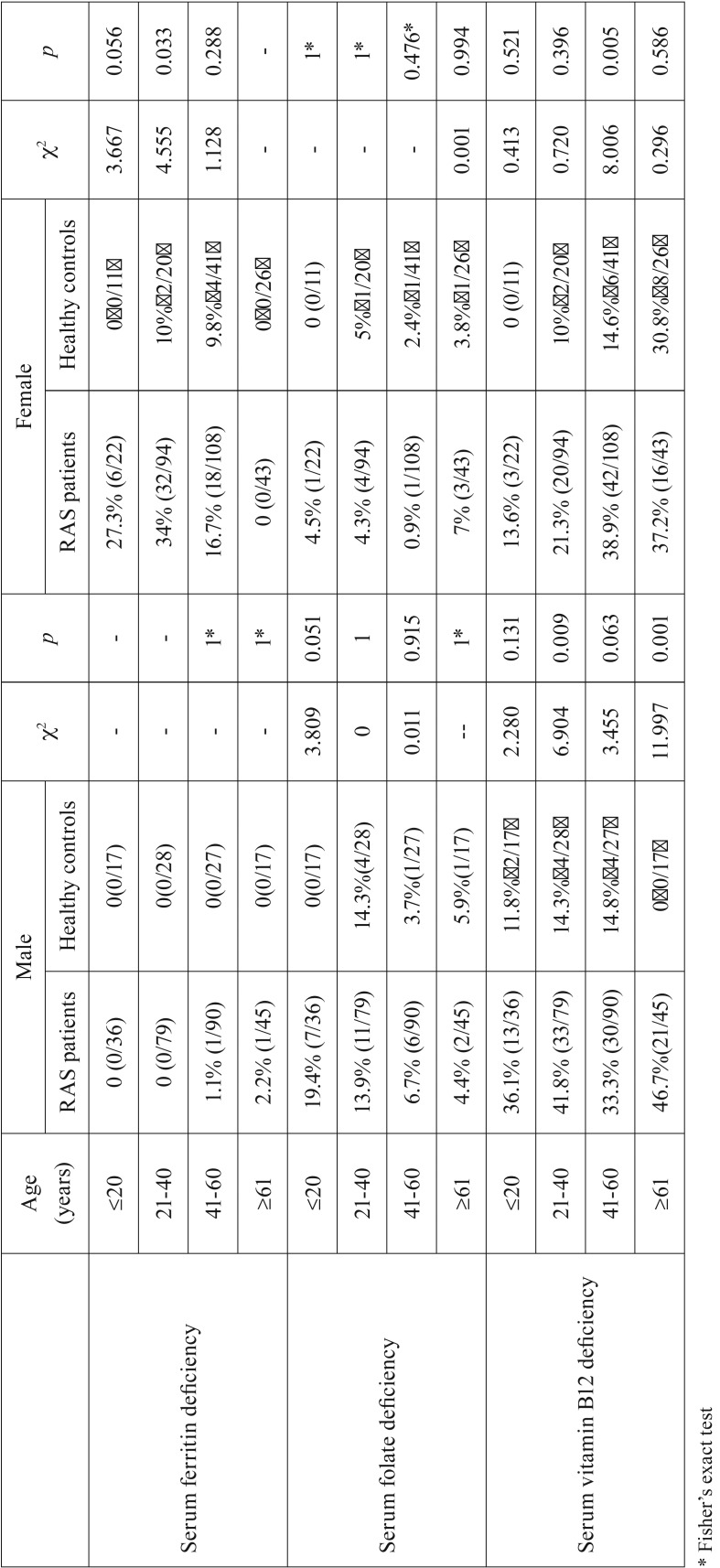
Hematinic deﬁciencies in RAS patients by gender and age compared with healthy controls.

Compared with healthy female controls, female RAS patients had a significantly higher frequency of serum ferritin deficiency in the young adult group (*p*=0.033), and a borderline significant difference in the adolescent group (*p*=0.056), but no significant differences were found in the middle-aged group or older group. Similar to the male group, no significant difference in rate of folate deficiency in each age group was found between female RAS patients and healthy female controls. Furthermore, female RAS patients had a higher frequency of serum vitamin B12 deficiency in each age group, but with a statistically significant difference in the middle-aged group only (*p*=0.005,  Table 3).

4. The correlation between the two factors (age and gender) and presence of hematinic deficiencies in the RAS patients group compared with that in the healthy control group.

In the RAS patients, both gender and age were validated as the significant variable associated with the hematinic deficiencies ( Table 4). By contrast, no significant statistical correlation was found between these two factors and the presence of hematinic deficiencies in the healthy control group ( Table 4).

**Table 4:**
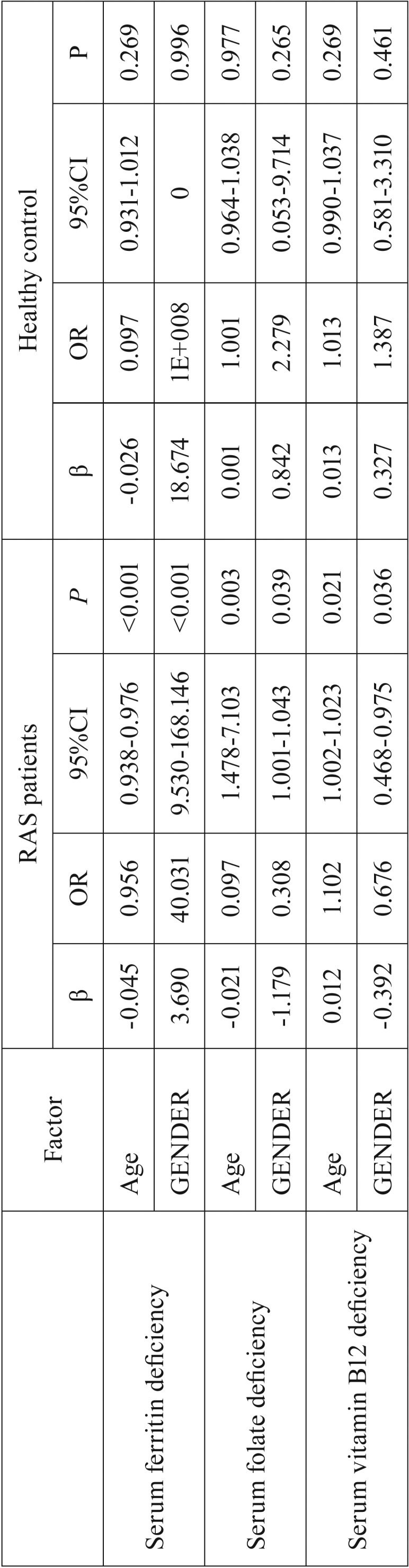
Logistic regression analysis on the correlation between the two factors (age and gender) and the presence of hematinic deficiencies.

## Discussion

Oral health is closely linked to the general health (21), with the oral cavity often revealing early signs and symptoms of certain underlying systemic diseases (22-24). RAS, the most common ulcerative disease of the oral mucosa, may be indicative of underlying hematologic conditions or nutritional disorders, including ferritin, folic acid, and vitamin B12 deficiencies (3,25). This observation was also demonstrated by our present study, which found evidences that RAS patients have a significantly higher frequency of hematinic deficiency than healthy control subjects. The precise mechanisms of how hematinic deficiencies affect RAS are still unknown, resulting in oral epithelial atrophy, injury to mucous membrane integrity and negative impact on the epithelial barrier were speculated as the primary mechanisms (16,23). Inadequate dietary intake, rather than the malabsorption, may be the main reason of hematinic deficiencies (12).

Compared with previous studies, advantages of this study were that gender and age were taken into account in a larger number of RAS patients, and that more accurate and comprehensive results were revealed. Similar to a number of previous studies (7,9), the preliminary results of our study show that only serum vitamin B12 deficiency was significantly different between the RAS patients and healthy controls, with neither serum ferritin deficiency nor serum folate acid deficiency shown to be related to RAS. However, these statistical findings were transformed after gender and age were taken into account. For example, a significantly higher frequency (close to one third) of serum ferritin deficiency was found in female RAS patients compared with female healthy controls, especially in the adolescent and young adult groups. This result indicates that serum ferritin deficiency may be much more common in young female RAS patients than previously assumed. Therefore, dentists should be aware that young female RAS patients may have a tendency to serum ferritin deficiency. Logistic regression analysis also revealed the significant correlations between the two factors (age and gender) and the presence of hematinic deficiencies (serum ferritin, folic acid, and vitamin B12 deficiencies) in the RAS patients. By contrast, such correlations were not found in the healthy controls. All these results strongly indicated that gender and age should not be ignored in related studies.

Two cases of borderline significance were observed in the adolescent group (age ≤20); in the comparison of serum ferritin deficiency in females (*p*=0.056) and in the comparison of serum folate deficiency in males (*p*=0.051). Furthermore, a large (but not significant) difference were observed in the same age group between the male RAS patients and the corresponding controls (36.1% vs 11.8%, *p*=0.131). Taken together, these results indicate that hematinic deficiencies are also common in adolescent RAS patients. Further studies are needed to investigate hematinic deficiencies in a larger cohort of this specific group of RAS patients.

Anemia and hematinic deficiencies were both considered as serious public health issues, especially in the developing countries (18), including China (26). RAS can be a “sentinel symptom”, anticipating the onset of anemia or hematinic deficiencies and allow for early diagnosis and treatment (11). Our present study also supports the view that routine hematological screening for hematinic deﬁciencies should be assessed in all patients with RAS (8,11,14). If a hematinic deficiency is involved, appropriate supplementation may resolve symptoms completely or reduce symptoms (11,27). Moreover, early supplementation may prevent the onset of important related systemic manifestations caused by ferritin, folic acid, or vitamin deﬁciency, to the benefit of the systemic health of RAS patients. Therefore, the study on the association between hematinic deficiencies and RAS is very worthwhile and may have a profound benefit for the public health.

Although hematinic deficiency has been validated as a predisposing factor in the etiopathogenesis of RAS in our present study and others (7,9,12,13,16), daily multivitamin supplementation for RAS patients is not recommended because of the poor efficacy of this approach (28). A rational management strategy for RAS should include routine hematological screening and corresponding specific replacement therapy when hematinic deficiencies are exhibited (1,27). If no hematinic deficiency is observed in mild RAS patients with oral ulcers, local symptomatic treatment, including various mouthwashes and topical agents, are the first option for decreasing symptoms (4). In this case, systemic medications are not recommended first line. If no hematinic deficiency is observed or the effect of replacement therapy for moderate and severe RAS patients is unsatisfactory, further investigations should be conducted into other predisposing factors such as immune disturbances, food hypersensitivity, endocrine alterations, and psychological evaluation (4-6,29). The administration of systemic medications was considered, but their potential side effects should be evaluated carefully (1,29).

The present study focuses on minor RAS, mainly because the large majority of patients with RAS suffer from the minor form and the pathogenesis of minor RAS may be somewhat different from other types of RAS (30). However, a recent study found no significant differences in the frequency of hematinic deficiencies between minor RAS and major RAS patients (16). Besides, one limitation of our study is that all participants were from single center. Further multicenter studies in larger cohorts are needed to verify our results, all clinical forms of RAS (minor, major and herpetiform ulcers), should also be investigated.

In conclusion, significant variations in hematinic deficiencies were demonstrated in RAS patients across different genders and age groups. We suggest that further studies on the hematinic deficiencies of RAS patients should be conducted, taking into account age and gender. Our findings in the present study may have important clinical implications for the management and treatment of RAS patients.
